# Novel Biobased Polyamide 410/Polyamide 6/CNT Nanocomposites

**DOI:** 10.3390/polym10090986

**Published:** 2018-09-04

**Authors:** Itziar Otaegi, Nora Aramburu, Alejandro J. Müller, Gonzalo Guerrica-Echevarría

**Affiliations:** 1 POLYMAT and Polymer Science and Technology Department, Faculty of Chemistry, University of the Basque Country UPV/EHU, Paseo Manuel de Lardizabal 3, 20018 Donostia-San Sebastián, Spain; itziar.otaegi@ehu.eus (I.O.); nora.aramburu@ehu.eus (N.A.); alejandrojesus.muller@ehu.es (A.J.M.); 2 IKERBASQUE, Basque Foundation for Science, 48013 Bilbao, Spain

**Keywords:** biopolymers, nanocomposites, conducting polymers, mechanical properties, structure-property relations

## Abstract

Biobased polyamide 410 (PA410)/multiwall carbon nanotube (CNT) nanocomposites (NCs) were obtained by melt-mixing in a twin screw extruder a Polyamide 6 (PA6)-based masterbatch (with 15 wt % CNT content) with neat PA410. Directly mixed PA410/CNT NCs were also obtained for comparison purposes. Transmision Electronic Microscopy (TEM) observation and conductivity measurements demonstrated that a good dispersion of CNTs was obtained, which was probably induced by the full miscibility between PA410 and PA6 (in the concentration range employed here), as ascertained by Differential Scanning Calorimetry (DSC) tests. As a result, the PA410/PA6/CNT NCs showed superior mechanical behaviour (≈10% Young’s modulus increase with a 4 wt % CNT content) than the binary PA410/CNT NCs (≈5% Young’s modulus increase with a 6 wt % CNT content), as well as superior electrical behaviour, with maximum conductivity values of approximately three orders of magnitude higher than in the binary PA410/CNT system, and lower percolation threshold values (0.65 wt % CNT content vs. 3.98 wt % CNT). The good dispersion and enhanced mechanical and electrical properties of these novel biobased nanocomposites, broadens their potential applications, such as electrical and electronics (E&E) or automotive industries.

## 1. Introduction

In recent years, there has been a growing need for the development of more sustainable products, and particularly, there is a demand for replacing petroleum-based polymers with plastics obtained from renewable resources. Importantly, the global production of biopolymers is expected to grow from 1.6 million metric tons in 2013 to 6.7 million metric tons in 2018 [1], and most of this growth is attributed to bio-based, non-degradable polymers rather than biodegradable polymers. Several factors are responsible for the increasing demand for bio-based polymers. Firstly, the production of bio-based polymers is one way of mitigating the negative impact of plastics on the environment. Secondly, with new technological developments it is possible to achieve a better price-performance ratio in the end product, thus expanding the fields of application. And finally, consumer demand for more sustainable products is also growing, and as a result, a wide variety of commercial biopolymers are currently available.

Polyamides (PA) are engineering polymers with very good thermo-mechanical properties and are widely used in the auto-parts, packaging, and electrical and electronics industries [2]. Polyamides from renewable resources have recently become available as it is now economically viable to produce bio-based diacids such as sebacic acid, a 10-carbon diacid derived from castor oil [1]. The PA410 used in this study, which has properties comparable to those of conventional technical polyamides such as PA6 or PA66, is made from bio-based sebacic acid [3]. Therefore, it has a renewable carbon content of about 70%, and its carbon footprint is reduced by almost 100% with respect to its competitors, which means that the amount of carbon dioxide emitted during the production of PA410 is offset by the amount of CO_2_ absorbed during plant growth. The 4-carbon diamine counterpart, butanediamine, is typically derived from petroleum. However, it can also be derived from commercially available bio-based succinic acid, and from the direct fermentation of sugars [1]. It is to be expected that future developments will lead to 100% renewable PA410 becoming available on the market. Therefore, with a view to tailoring the specific properties of these materials, research into their blends and nanocomposites is of great interest now and in the future.

The study of polymer nanocomposites (NCs) based on carbon nanotubes (CNTs) has attracted significant academic and industrial interest in recent years, helped by the continuous optimization of their producing methods [4,5]. One of the most important contributions of CNTs in the field of polymeric matrices is that they enable electrically conductive materials to be obtained [6]. Conductivity is achieved when a three-dimensional network of interconnected nanotubes is formed within the matrix, which occurs upon reaching the so-called electrical percolation concentration (p_c_). Moreover, it is well known that the addition of CNTs improves the mechanical, thermal and barrier properties of polymeric materials. Therefore, PA/CNT NCs are of great interest from an engineering point of view, and have been widely studied in the literature. PA6 is by far the most widely used commercial polyamide in the fabrication of PA/CNT-based NCs [7,8,9], but other PAs such as PA66 [10,11], PA12 [12], PA610 [13], PA1010 [14] or PA11 [15], and less frequently PA46 [16] or PA1212 [17] have also been studied. In these works, the thermal properties of NCs were studied, as well as the effect on the dispersion of CNTs and the mechanical and electrical properties of the NCs of different aspects such as the processing conditions, the condition of the CNTs (pristine or modified), their aspect ratio, and the viscosity of the matrix.

Commercially viable polymer/CNT-based NCs are generally obtained when efficient dispersion of the CNTs is achieved, and conventional melt mixing [18,19] is preferred over other methods of production because it is fast, simple, solvent-free and does not require specific equipment [20]. However, due to the usually high viscosity of the polymeric matrices, the CNT-dispersion level attained is seldom comparable to that obtained from solution or “in situ” polymerization methods. For this reason, well-dispersed masterbatches (usually in the 10–20 wt % CNT content range), pre-produced in ad hoc machinery, have often been employed as they enable homogeneous dispersions to be obtained. PA-based masterbatches are commercially available. For instance, Jiang et al. [13] studied PA610/CNT NCs obtained from a masterbatch containing 8 wt % CNT. The significant reduction in the thermal expansion coefficient and the improved electrical properties with an electrical percolation concentration in the range between 2–6 wt % were ascribed to the formation of a 3D CNT network. Meincke et al. [21] studied PA6/CNT NCs (obtained from a masterbatch with 20.3 wt % CNT) and observed an electrical percolation threshold in the range between 4–8 wt %. All samples showed an increased Young’s modulus with increasing filler content, showing a 45% increase with 6 wt % CNT. 

When a specific matrix-based masterbatch is not commercially available, blending that matrix with another polymer-based masterbatch, as long as the latter polymer is miscible or compatible with the former, has proved an effective means of obtaining good CNT dispersion levels. Gonzalez et al. [22] diluted a polybutylene terephthalate (PBT)/CNT masterbatch in polyetherimide (PEI) in order to obtain high performance PEI-based nanocomposites. They confirmed that PEI and PBT were fully miscible and observed that the nanotubes were efficiently dispersed in the PEI-rich matrix, mostly in the form of single nanotubes. They obtained a p_c_ close to 1 wt %, clearly lower than that observed in highly dispersed PEI-based NCs using the ultrasound-assisted melt processing method (2 wt %) [23]. Furthermore, the increase observed in the modulus was similar in both cases. Arboleda-Clemente et al. [2] studied the effect of the PA ratio and the masterbatch used, PA66/CNT or PA6/CNT, on the CNT dispersion of PA66/PA6/CNT NCs. They attributed the higher conductivity values obtained with the 50/50 PA66/PA6 CNT composites to the greater degree of miscibility between the PAs in the composition, among other factors. 

In the present work, with the aim of obtaining bio-based PA410/CNT NCs which, to the best of our knowledge, have not been previously studied, a commercial PA6-based masterbatch (15 wt % CNT content) was diluted with neat PA410. The nanostructure, phase behaviour, mechanical, and electrical behaviour were studied and, given the importance of the compatibility/miscibility of the two PAs, the phase behaviour of the unfilled PA410/PA6 system in the composition range studied here is also discussed.

## 2. Materials and Methods

The PA410 used in this work was EcoPaXX^®^ Q150-D, kindly provided by DSM (Genk, Belgium), while the PA6 used to evaluate the behaviour of unfilled PA410/PA6 blends was Durethan^®^ B30S, provided by Lanxess (Cologne, Germany). A PA6/CNT masterbatch (containing 15 wt % CNT) manufactured by Nanocyl (Sambreville, Belgium) and commercialized as Plasticyl^®^ PA 1503, was also used. The CNTs used in this masterbatch are MWCNTs (Nanocyl NC7000), with an outer diameter of 9.5 nm, average length of 1.5 μm and a carbon purity of 90%. It should be noted that the PA6 employed by Nanocyl to manufacture their commercial masterbatch is probably not the same as the PA6 Durethan^®^ B30S.

In order to prevent moisture-induced degradation reactions during processing, PA410, PA6 and the Plasticyl^®^ PA 1503 masterbatch were dried in a dry air dehumidifier (Wittmann Drymax, Kottingbrunn, Austria) for 48–72 h at 80 °C. 

PA410/PA6/CNT (CNT content ranging from 0.5 to 4 wt %) NCs were obtained by melt-mixing using a Collin ZK25 co-rotating twin screw extruder-kneader (Ebersberg, Baviera, Germany) at 270 °C with a screw rotation speed of 200 rpm by diluting the Plasticyl^®^ PA 1503 masterbatch with the adequate amounts of neat PA410. The diameter and length-to-diameter ratio of the screws were 25 mm and 30, respectively. Directly melt-mixed PA410/CNT binary NCs were also obtained under the same conditions, for comparison purposes. In this case, pristine multi-walled carbon nanotubes, with an outside diameter of 20–30 nm and purity higher than 95%, provided by Cheaptubes, were employed. At this point, it must be assumed that the comparison between both systems will also be influenced by the different nature of these CNTs and those of the masterbatch. Table 1 shows the wt % content of each component for all the compositions studied.

The reference unfilled PA410/PA6 blends (prepared employing EcoPaXX^®^ Q150-D and Durethan^®^ B30S materials), were obtained in the same equipment with the same processing conditions in a composition range of 0–25 wt % PA6 and are included in Table 1.

The commercial PA6/CNT masterbatch supplied by Nanocyl employs a different PA6 matrix than that of the PA6 homopolymer Durethan^®^ B30S, which was employed to prepare the reference unfilled blends. Given the unknown intrinsic characteristics (molecular weight, viscosity, crystallinity...) of the PA6 employed in the manufacture of the commercial CNT masterbatch, DSC experiments were conducted to at least compare the thermal behaviour of the masterbatch and that of the reference PA6 selected to prepare the unfilled PA410/PA6 blends. In Figure S1 and Table S1 (Supplementary Material), it is shown that before processing both PA6 samples exhibit a similar calorimetric behaviour, without any significant difference in their melting points. This could be expected as they are both injection moulding grade samples. It is very difficult to determine the molecular weight of the PA6 within the masterbatch sample, as removing all CNTs by extraction is nearly impossible.

Although a deep rheological characterization lies out of the scope of this work, due to the importance of the matrix viscosity in the dispersion level of the CNTs in any system, the torque during melt mixing, under the employed processing conditions, was measured in a DSM Micro 5cc Twin Screw Compounder using recirculation mode and 5 min time, for neat PA410 and for the PA410/PA6 blend with the maximum PA6 content (75/25). The values obtained were 610 ± 40 N and 660 ± 50 N respectively, clearly indicating that the matrix viscosity is very similar in both cases.

The extrudates were cooled in a water bath (20 °C), pelletized, and dried again. Subsequent injection moulding of dried pellets was carried out in a Battenfeld PLUS 350/75 reciprocating screw injection moulding machine (Kottingbrunn, Austria) with a press closing force of 350 kN to obtain tensile (ASTM D-638, type IV, thickness 2 mm) specimens. The screw of the plastization unit had a diameter of 25 mm and an L/D ratio of 14. The melt and mould temperatures were 270 °C and 85 °C, respectively. The injection speed, pressure-holding time and cooling time were 42 cm^3^/s, 3 s and 15 s, respectively. All specimens were kept in a desiccator to avoid post-processing humidity absorption. 

Standard circular sheets for electrical conductivity measurements (diameter and thickness: 70 mm and 1 mm, respectively) could only be obtained by hot pressing in a Collin P200E hydraulic press (Ebersberg, Baviera, Germany). The moulding process was developed at a temperature of 270 °C and a pressure of 130 bar in three stages: preheating or plasticizing (closure without pressure, 2 min), compression (closure under pressure, 3 min), and cooling under pressure (6 min). Therefore, conductivity is the only property of the materials prepared in this work that was not measured directly in injection moulded tensile specimens. It can be argued that a different CNT dispersion would be expected from both processing techniques (injection moulding and hot pressing), but the methodology remains valid when the objective of these measurements is to compare the electrical conductivity of the materials obtained with and without PA6-based masterbatch.

The phase behaviour was studied by DMA using a TA Q800 viscoelastometer that provided the loss tangent (tanδ) against temperature. The scans were carried out in single cantilever bending mode at a constant heating rate of 4 °C/min and a frequency of 1 Hz, from −100 °C to 150 °C. The melting and crystallization behaviour of the materials were studied by DSC using a Perkin-Elmer DSC-7 calorimeter (Waltham, MA, USA) calibrated with reference to an indium standard. The samples were first heated from 30 °C to 300 °C at 20 °C/min and then cooled at the same rate. A subsequent second heating scan was also performed on unfilled PA410/PA6 blends in order to better understand their phase behaviour. The melting and crystallization temperatures (*T_m_*, *T_c_*) were determined, respectively, from the maxima of the corresponding peaks during the heating and cooling scans, and the melting and crystallization enthalpies were determined from the areas of each of these peaks. The degree of crystallization of PA410 was calculated from the melting and cold crystallization enthalpies, taking the enthalpy of a 100% crystalline (Δ*H_f_^∞^*) PA410 as 269 J/g [1].

The nanostructure was analyzed by Transmission Electron Microscopy (TEM). The samples were obtained from injection moulded specimens and ultrathin-sectioned at ~100 nm using a Leica EMFC 6 ultramicrotome equipped with a diamond knife. The micrographs were obtained in a Tecnai G2 20 twin apparatus (FEI, Waltham, MA, USA), operating at an accelerating voltage of 200 kV.

The tensile tests were carried out in an Instron 5569 tensile tester (Instron, Norwood, MA, USA). Young’s modulus was determined by means of an extensometer at a crosshead speed of 1 mm/min. Tensile strength (σ_t_) and ductility, measured as the break strain (ε_b_) were determined from the load-displacement curves at a crosshead speed of 10 mm/min. A minimum of five tensile specimens were tested for each reported value.

The electrical resistivity was determined according to the ASTM D4496-87 standard. Volume resistances were measured and converted to conductivity values. Measurements were taken at 10V using a Keithley 6487 picoammeter (Cleveland, OH, USA) and a Keithley 8009 Resistivity Test Fixture.

## 3. Results and Discussion

### 3.1. Phase Behaviour

#### 3.1.1. Unfilled PA410/PA6 Blends

**Figure 1:**
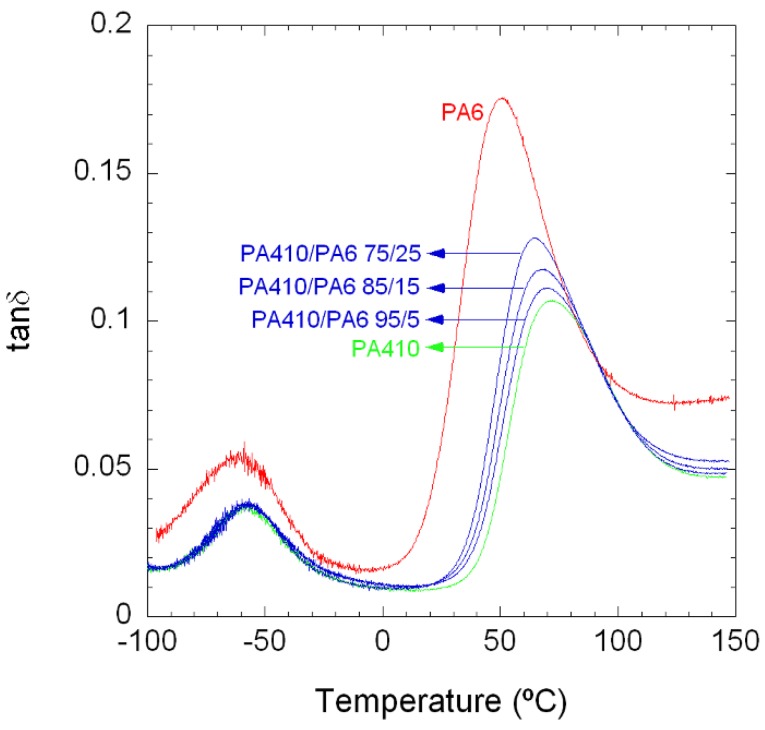
DMTA curves of neat PA410, PA6, and 95/5, 85/15, and 75/25 PA410/PA6 blends.

Figure 1 shows the tanδ vs. temperature curves of neat PA410, PA6, and their PA410/PA6 blends up to a 25% PA6 content. As can be seen, both PAs and all the blends show two peaks, which correspond to the α (high temperature peak) and β (low temperature peak) relaxations [24,25]. The α relaxation is the mechanical manifestation of the glass transition temperature or *T*_g_. The β relaxation hardly changes with blend compositions and is almost the same for both neat PA6 and neat PA410. This low temperature transition is known to be related to local range motions within the chains which are very similar for the two types of polyamides employed here and their blends.

**Figure 2:**
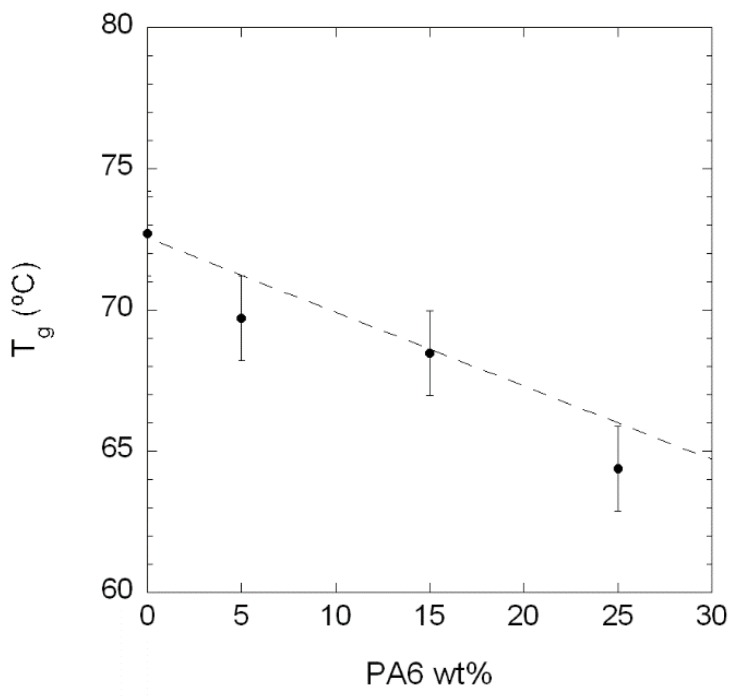
Glass transition temperature (*T*_g_) of the PA410/PA6 blends vs. PA6 content. The dotted line corresponds to the Fox equation for miscible blends [26].

All blends show a single *T*_g_ peak, located at intermediate temperatures between those of neat PA410 (72.7 °C) and neat PA6 (51.6 °C). Figure 2 shows *T*_g_ values (taken as the peak position of the α relaxation in Figure 1) as a function of PA6 content. The *T*_g_-values are well predicted by the Fox equation for miscible blends [26], represented by the dotted line in Figure 2. This behaviour is indicative of full PA410/PA6 miscibility in the amorphous phase within the composition range studied.

**Figure 3:**
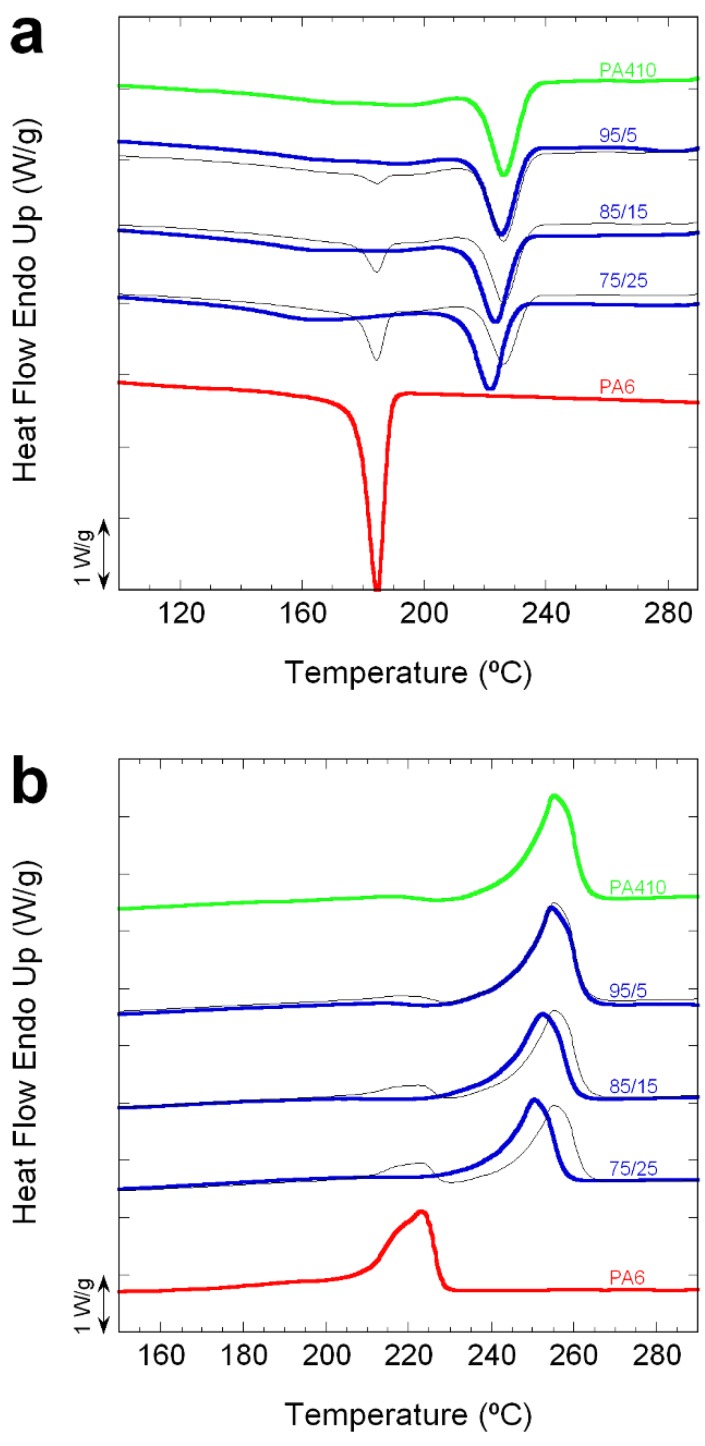
Normalized experimental (thick lines) crystallization (**a**) and melting (**b**) DSC curves of PA410, PA6, and 95/5, 85/15 and 75/25 PA410/PA6 blends. Thin black lines correspond to the calculated curves (assuming full immiscibility between both PAs). Curves have been shifted in the vertical axis.

With respect to the crystalline phase of the blends, Figure 3a,b show the DSC curves of the cooling and subsequent heating scans of both neat PAs and all the blend compositions studied. The main calorimetric data (*T_m_*, *T_c_*, Δ*H_m_*, Δ*H_c_*) of these curves, as well as those corresponding to the first heating scans, is presented on Table 2. Additionally, “unmixed” DSC traces were generated mathematically (i.e., using a simple mixing law based on the experimental traces of the neat components). The DSC traces of “unmixed” blends represent what the thermal response of the materials would be if they were completely immiscible.

As Figure 3a,b shows, in the case of neat PA6, a single crystallization and melting peak appears at 185 °C and 223 °C, during cooling and heating, respectively. However, neat PA410 presents bimodal crystallization and melting, more evident in the former than in the latter, with a narrow main peak at 226 °C and 255°C, respectively, and a broad and undefined peak at lower temperatures.

**Figure 4:**
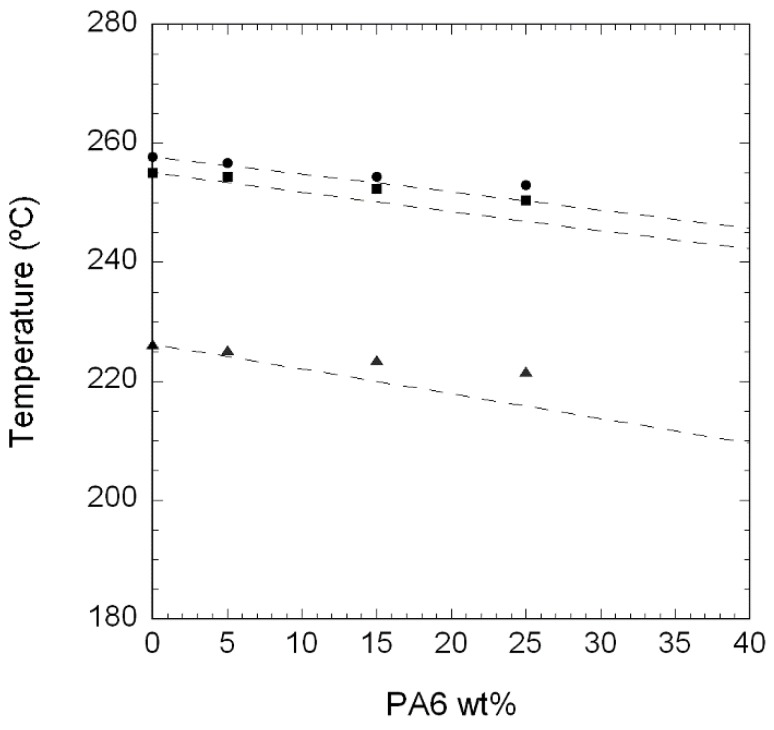
*T_c_* (▲) and *T_m_* values (first (●) and second (■) heating scans) of neat PA410 and PA6 and of 95/5, 85/15 and 75/25 PA410/PA6 blends.

Regarding the blends, all the compositions showed crystallization and melting behaviour similar to that of neat PA410 (see Figure 3a,b). This is because bimodal crystallization and melting can also be observed in the three compositions studied, with no apparent sign of separate PA6 crystallization or melting. However, although the main narrow exothermal peak in the blends appeared close to that of neat PA410, its position moved towards lower temperatures as the PA6 content increased. This decrease can be clearly observed in Table 2 and Figure 4, which shows the *T_c_* and *T_m_* (first and second heating scan) vs. PA6 content. As previously mentioned, and although it does not accurately fit a simple rule of mixtures between neat components (marked by the dotted lines), a progressive decrease in both *T_m_* and *T_c_* can be observed as the PA6 content increases in Figure 4.

Although the overall thermal behaviour points to the co-crystallization of both PAs in the blends, it is still not possible to rule out a separate crystallization of a small amount of PA6 because the second broad and undefined crystallization and melting peak of neat PA410 appears at the same temperatures at which PA6 would, in theory, crystallize and melt. This is why the normalized experimental crystallization and melting DSC curves were compared with calculated ones (assuming full immiscibility between both components, or “unmixed” blends) in Figure 3. As can be seen, the neat PA6 corresponding crystallization and melting peaks that should appear in the blends if separate crystallization and melting occurred (thin black lines in Figure 3 in the temperature range corresponding to neat PA6), cannot be observed in the experimental curves, and thus the co-crystallization of both PAs in the blends, in the range studied, is a strong possibility. Wide angle X-ray studies as a function of temperature would be needed to confirm co-crystallization and/or the presence of two phases (e.g., a PA6 rich phase and a PA410 rich phase). However, such experiments are beyond the scope of the present paper. 

With respect to the crystallization and melting enthalpies, given the very similar values of both neat PAs (Table 2), no significant conclusions regarding the blends can be obtained from these data.

Summarizing the results of this section, both the DMA and the DSC indicate that PA6 and PA410 are probably fully miscible in the amorphous state and may also show mixed crystalline phases (i.e., PA6 and PA410 may be able to co-crystallize). 

#### 3.1.2. PA410/PA6/CNT NCs

**Figure 5:**
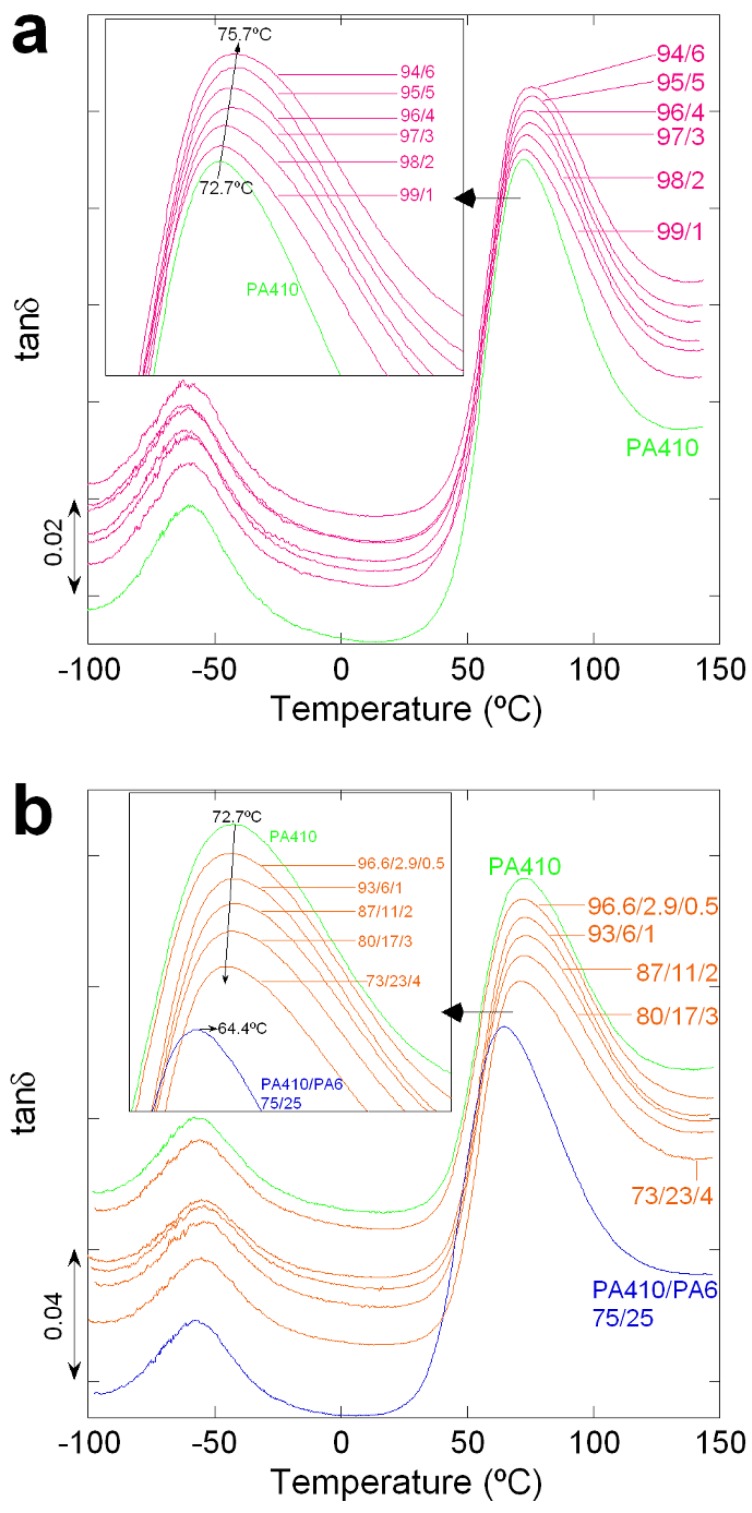
DMTA curves of (**a**) neat PA410 and PA410/CNT NCs and (**b**) neat PA410, the 75/25 PA410/PA6 blend and PA410/PA6/CNT NCs. Curves have been shifted in the vertical axis.

Figure 5 shows the tanδ vs. temperature curves of neat PA410 and PA410/CNT NCs (a) and of PA410/PA6/CNT NCs (b). As can be seen in Figure 5a, the two peaks which appear in neat PA410 can also be observed in the PA410/CNT NCs. When the position of the *T*_g_ peak of neat PA410 is compared with that of the PA410/CNT NCs, the *T*_g_ is seen to increase slightly with increasing CNT content (up to 3 °C in the 94/6 composition). In the literature, this behaviour has been attributed to the hindering effect of the CNTs in the movement of polymeric chains in the amorphous phase and to the reduction of the free volume which has also been observed in other PA/CNT NCs [7,8,27].

With respect to the ternary PA410/PA6/CNT NCs, in Figure 5b the corresponding *T*_g_ peaks appear at slightly lower temperatures than that of neat PA410 (72.7 °C) as the CNT content increased (for example, 71 °C for the 73/23/4 composition). While close to the experimental error of the measurements, in the light of the results mentioned in Figure 1 and Figure 2, this decrease can be attributed to the presence of miscible PA6 whose content increased with the CNT content. Thus, the position of the *T*_g_-peak of the PA410/PA6/CNT NCs is the result of two opposing effects, the increased mobility of the polymeric chains in the amorphous phase caused by the miscible PA6 component, and the mobility hindering effect of the PA410/CNT components, due to the interactions with CNTs.

**Figure 6:**
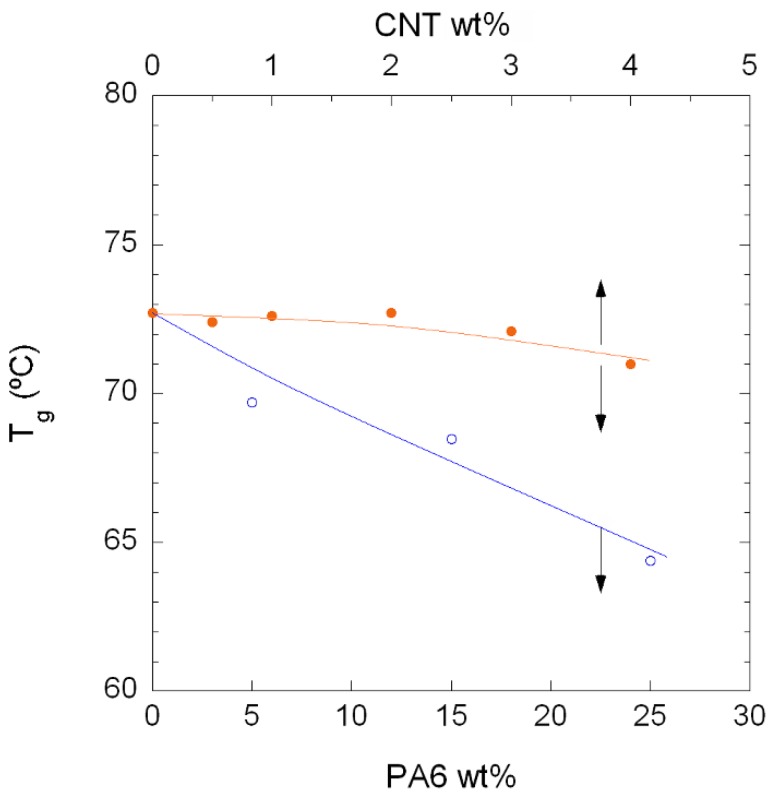
Glass transition temperature (*T*_g_) of the unfilled PA410/PA6 blends (○) and PA410/PA6/CNT NCs (●) vs. CNT and PA6 content.

In order to observe the combined effect of the PA6 and the presence of the CNTs in greater detail, Figure 6 shows the *T*_g_ values of the unfilled PA410/PA6 blends (open symbols) and the PA410/PA6/CNT NCs (filled symbols) vs. the PA6 and CNT content. As the figure shows, the vertical distance between the two lines increases as the CNT content increases, indicating that the decrease in *T*_g_ caused by the PA6 in the NCs was mostly offset by the restricted mobility of the polymer chains around the CNTs. The overall thermal behaviour of the NCs can be considered positive, because even at the highest PA6 content (23%), the *T*_g_ of the corresponding 73/23/4 NC showed a limited 1.7 °C decrease with respect to the neat PA410.

Table 3 and Table 4 show, respectively, the calorimetric parameters (melting temperature (*T_m_*), enthalpy (Δ*H_m_*), and degree of crystallinity (χ_c_) of the first heating scan, which represents the thermal properties of the as-moulded materials, and the crystallization temperature (*T_c_*) and enthalpy (Δ*H*_c_) of the cooling scan) of the binary PA410/CNT and ternary PA410/PA6/CNT NCs.

As can be observed in Table 3, hardly any significant changes were observed in both the *T_m_* and χ_c_ of the binary PA410/CNT NCs, regardless of the CNT content, suggesting, as expected, that the CNTs had no effect on the melting behaviour of the PA. This has been reported for other PA/CNT NCs [7,9,12,28,29]. However, the *T_c_* and Δ*H_c_* increased to significantly higher values in comparison to the neat PA410. This is indicative of a nucleating effect caused by the CNTs, which enhances the overall non-isothermal crystallization rate of the PA410 from the melt. This has also been reported for other PA-based NCs [8,10,12,13,28,30].

With respect to the ternary PA410/PA6/CNT NCs, they showed single melting and crystallization peaks, as did the unfilled PA410/PA6 blends. As Table 4 shows, a progressive decrease of *T_m_* was observed at increasing CNT (and subsequent PA6) contents. This decrease was similar to that observed in the unfilled blends (Figure 4 and Table 2), pointing to the presence of PA6 as the cause of this behaviour and confirming that the CNTs had no effect on the melting behaviour of the PA matrix, in neither the neat PA410 nor in the miscible PA410/PA6 blend.

With respect to the *T_c_* and Δ*H_c_* of the PA410/PA6/CNT NCs, as in the case of the binary PA410/CNT NCs, they increased to higher values than those of neat P410, confirming the aforementioned nucleating effect of the CNTs on the crystallization process of the PA matrix from the melt. Moreover, the increase in *T_c_* (of 8–9 °C) is significantly lower than the increase observed in the binary PA410/CNT NCs (10–12 °C). This could be due to a combined effect of the nucleating behaviour of the CNTs and the drop in T_c_ caused by the presence of PA6 in the PA410 matrix (see Figure 4 and Table 2).

### 3.2. Nanostructure

**Figure 7:**
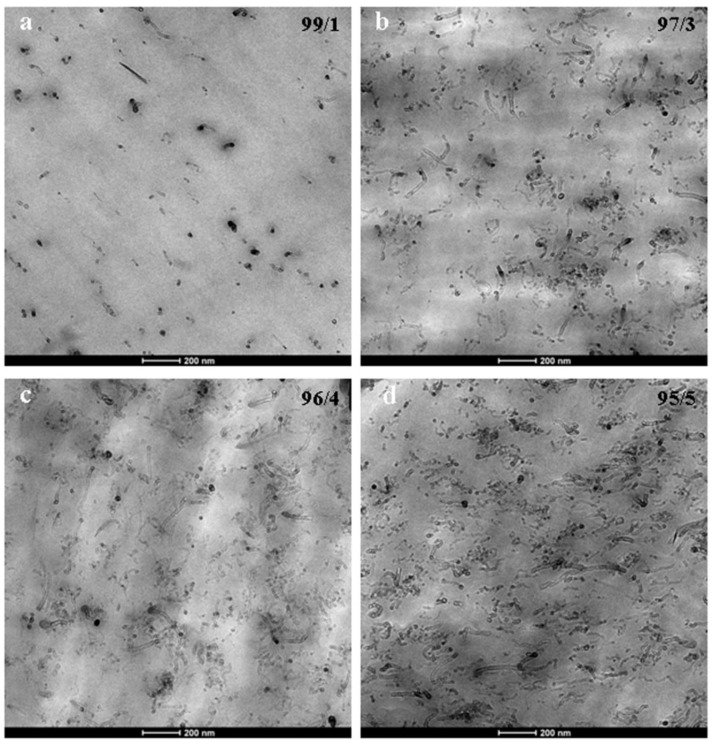
TEM micrographs of the 99/1 (**a**), 97/3 (**b**), 96/4 (**c**) and 95/5 (**d**) binary PA410/CNT NCs at 25,000× magnification.

**Figure 8:**
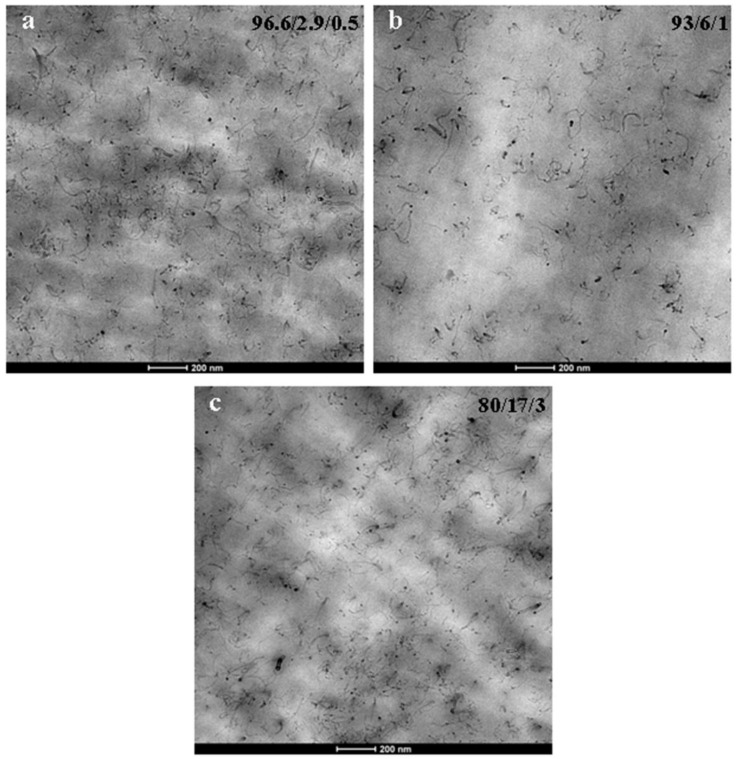
TEM micrographs of the 96.6/2.9/0.5 (**a**), 93/6/1 (**b**) and 80/17/3 (**c**) ternary PA410/PA6/CNT NCs at 25,000× magnification.

Figure 7 and Figure 8 show, respectively, the TEM micrographs of the binary PA410/CNT (99/1, 97/3, 96/4 and 95/5 compositions) and ternary PA410/PA6/CNT (96.6/2.9/0.5, 93/6/1 and 80/17/3 compositions) NCs. The micrographs indicate good dispersion of the CNTs in both the neat PA410 and the PA410/PA6 blend. The CNTs are uniformly distributed in the polymeric matrix and both individual CNTs and small aggregates can be observed, regardless of the CNT content. When binary PA410/CNT and ternary PA410/PA6/CNT NCs are compared, a systematic observation of all the TEM images points to a general increase in the degree of dispersion of the CNTs in the ternary NCs with respect to the binary ones, even when the differences are not clear from the images provided in Figure 7 and Figure 8, given the good dispersion level observed in both cases. As electrical conductivity is very sensitive to the dispersion level of CNTs in the matrix, it was measured and compared for both systems.

**Figure 9:**
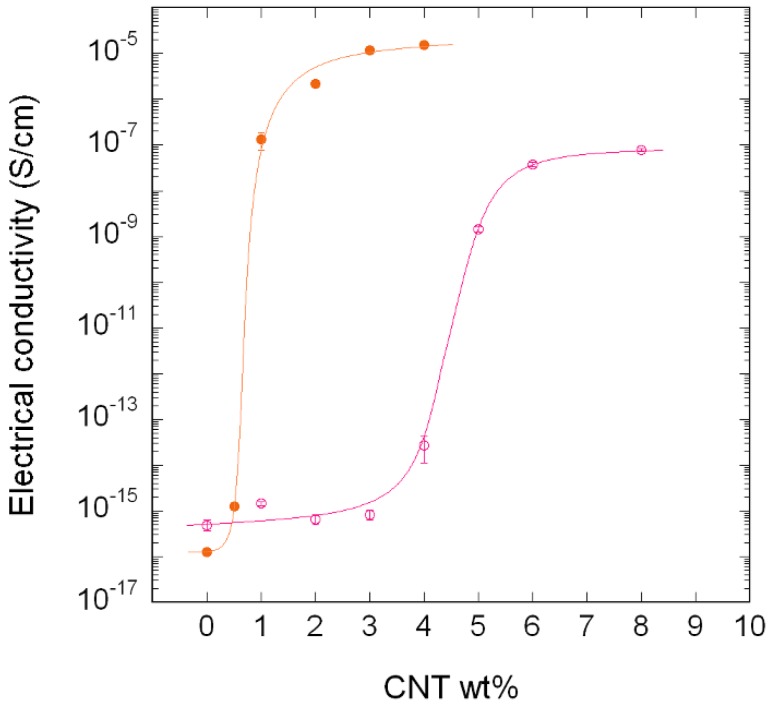
Electrical conductivity of PA410/CNT NCs (○) and of PA410/PA6/CNT NCs (●).

Figure 9 shows the electrical conductivity of PA410/CNT and PA410/PA6/CNT NCs vs. CNT content. As mentioned in the experimental section, specimens for electrical conductivity measurements were obtained by hot pressing instead of injection moulding, as was the case of the tensile specimens used for the characterization of all the other properties. However, both binary and ternary NCs samples employed for conductivity measurements have the same processing and post-processing history (i.e., extrusion followed by compression moulding). Thus, although a somewhat different CNTs dispersion could have been obtained as compared to that in the injection moulded samples, the conductivity results are still valid for comparison purposes between both systems.

As can be seen in Figure 9, the conductivity changed in both cases by over 7–8 decades, indicating that electrical percolation of the filler took place. However, when the curves of both systems are compared, the maximum conductivity value obtained is clearly higher (namely, three orders of magnitude), and electrical percolation took place at significantly lower CNT contents in the ternary PA410/PA6/CNT NCs than in the binary PA410/CNT NCs. The percolation threshold was calculated using a simple power law [31] and the values obtained were 3.98 wt % for the PA410/CNT binary system and 0.65 wt % for the PA410/PA6/CNT ternary system.

When attempting to ascertain the cause of the difference in behaviour between the binary and ternary NCs, orientation can be ruled out from the main factors known to affect conductivity in CNT-filled NCs. The TEM observations clearly indicate randomly oriented CNTs in both cases. With respect to the aspect ratio of the CNTs, the different nature of the CNTs employed in binary and ternary NCs was explained in the experimental section and, although it will play a significant role, it is not easy to separate its effect from that of the dispersion level. However, the importance of this factor must only be considered when similar dispersion levels of the CNTs are obtained. In addition, both binary and ternary NCs were obtained after similar processing and thus, the level of applied shear stress is also identical. Shear stress is known to significantly decrease the aspect ratio of the CNTs in melt processed NCs [29,32]. Therefore, assuming the different aspect ratio of the CNTs employed, only an improved dispersion level of the CNTs in the PA410/PA6/CNT NCs can account for the enhanced electrical behaviour of this system, providing additional support and confirming the TEM observations stated above. This behaviour must therefore be a consequence of (1) the very good CNT dispersion level that commercial masterbatches usually offer and (2) the full miscibility in the melt state of PA410 and PA6 which led to a similarly good dispersion level in the ternary NCs.

Table 5 shows the percolation thresholds obtained for PA-based systems in this and other works. Very different values, ranging from 0.4% to 6%, have been obtained depending on the matrix, processing method and conditions used. Regarding the NCs in this study, the value obtained for the binary PA410/CNT system is situated among the higher range of values reported in the literature, while that obtained for the ternary PA410/PA6/CNT system is one of the lowest values in the table. Among the many data available in the literature, and taking into account that PA410-based NCs have not been studied so far, to our knowledge, the data shown on Table 5 were selected from several melt processed PA matrices. Any discussion concerning the main parameters which affect the p_c_ value, i.e., orientation, aspect ratio, and dispersion level of the CNTs, would not be relevant given the different nature of the matrix and the CNTs in each study. However, Table 5 shows that the processing route proposed in this work (diluting a PA6/CNT masterbatch in PA410) is a very effective means of obtaining highly conductive bio-based PA/CNT NCs with p_c_ values among the lowest reported in the literature for PA-based NCs.

### 3.3. Mechanical Properties

**Figure 10:**
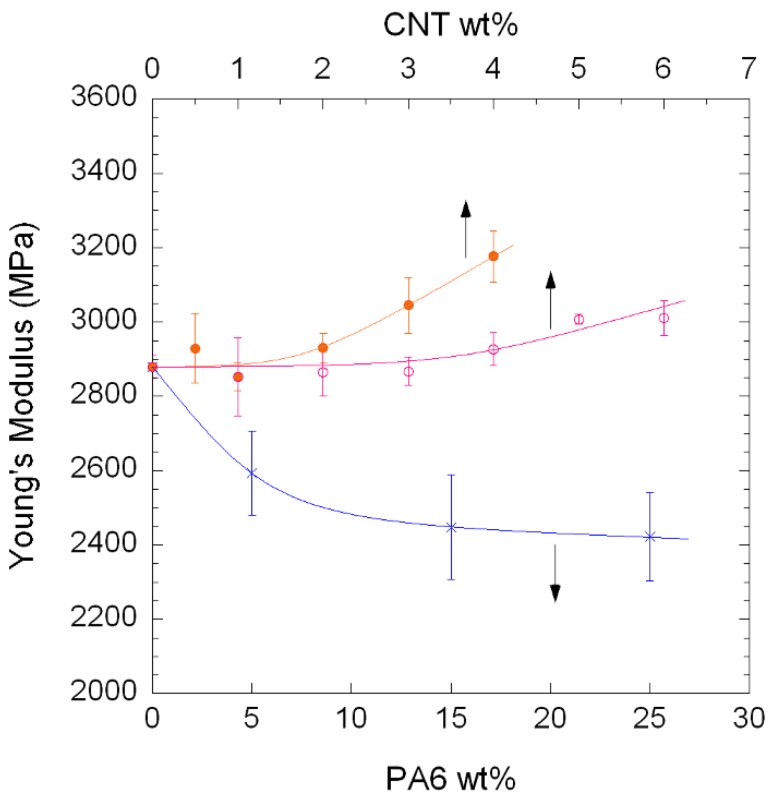
Young’s modulus of PA410/CNT NCs (○), PA410/PA6/CNT NCs (●), and unfilled PA410/PA6 blends (x).

Table 6 shows Young’s modulus, tensile strength, and ductility values of both ternary and binary NCs. The behaviour of Young’s modulus of both the binary PA410/CNT (open symbols) and ternary PA410/PA6/CNT NCs (filled symbols) vs. the CNT and PA6 content is also shown in Figure 10. As both CNTs and PA6 content influence the mechanical behaviour of the ternary NCs, Young’s modulus of the unfilled PA410/PA6 blends is also shown as a reference. As to be expected in well-dispersed CNT-filled NCs, the stiffness increased at increasing CNT contents in both NC systems. This increase is a consequence of a decrease in the molecular mobility of the polymeric chains indicated by the *T*_g_ increase shown by DMTA. The large interfacial area-to-dispersed phase volume ratio characteristic of well-dispersed CNTs facilitates the presence of interactions between the polymer and filler [30]. Moreover, when binary PA410/CNT and ternary PA410/PA6/CNT systems are compared, the modulus increase is significantly higher in the latter (10.3% increase for a 4 wt % CNT content) than in the former (4.5% increase for a 6 wt % CNT content). 

The superior mechanical behaviour of the ternary PA410/PA6/CNT NCs took place even when Young’s modulus for PA6 (≈2400 MPa) was significantly lower than that of neat PA410 (≈2900 MPa). When the modulus increase in the PA410/PA6/CNT NCs is calculated with respect to the unfilled PA410/PA6 blend, this increase reaches 30% for the 73/23/4 composition. As previously mentioned for conductivity, the most important factors that affect mechanical behaviour of CNT-filled NCs are orientation, aspect ratio, and dispersion level. For the reasons discussed above, the random orientation of the CNTs in both cases rules out this factor and the combination of the different aspect ratio and superior dispersion level of the ternary NCs, whose separate contribution would not be easy to evaluate, accounts for the outstanding Young’s modulus results.

The yield stress reported in Table 6 did not significantly change as the CNT content increased, with respect to that of neat PA410 (≈75 MPa) in the binary PA410/CNT or the ternary PA410/PA6/CNT NCs. Although positive tensile strength behaviour—similar to that of Young’s—has been observed in CNT-filled NCs [8,12,28], the local nature of the yielding process—in contrast to Young’s modulus where the whole section of the specimen contributes—usually leads to constant [7,36] or even decreasing [7,37] values as the CNT content increases. However, taking into account that the yield stress value of neat PA6 (66 MPa) is significantly lower than that of PA410, the constant values of the tensile strength in the ternary PA410/PA6/CNT NCs can be considered positive behaviour.

The ductility of the binary PA410/CNT and ternary PA410/PA6/CNT systems sharply decreased even at the lowest CNT contents, as is to be expected in CNT-reinforced NCs. This decrease is attributed to restrictions in the mobility of the matrix chains caused by the single CNTs, which promotes fracture, and to the intrinsic crack-sensitive nature of PAs [38,39,40]. No relevant differences were observed between them, and all the filled compositions broke just after yielding.

## 4. Conclusions

PA410/CNT NCs with enhanced mechanical and electrical behaviour were obtained by an alternative route to direct melt-mixing of the two components in a twin-screw extruder. The use of a PA6/CNT masterbatch (CNT wt % content = 15%) led to a good dispersion level of the nanotubes in the PA410 matrix, aided by the full miscibility of PA410 with PA6 (in the concentration range employed). The ternary PA410/PA6/CNT NCs showed a single *T*_g_, which shifted only marginally to lower values, even when the *T*_g_ of neat PA6 was significantly lower. The increases in Young’s modulus observed at increasing CNT contents in the ternary NCs were more than twice those of the binary NCs. Moreover, the maximum electrical conductivity values were three orders of magnitude higher in the PA410/PA6/CNT NCs than in the PA410/CNT NCs and the percolation threshold decreased from 3.98 to 0.65 CNT wt %.

## Figures and Tables

**Table 1:** PA410, PA6 and CNT wt % content in the compositions studied.

	Nomenclature	PA410 (wt %)	PA6 (wt %)	CNT (wt %)
PA410/ CNTbinary NCs	100/0	100	-	0
	99/1	99	-	1
	98/2	98	-	2
	97/3	97	-	3
	96/4	96	-	4
	96/5	95	-	5
	94/6	94	-	6
PA410/PA6/CNT ternary NCs	96.6/2.9/0.5	96.6	2.9	0.5
	93/6/1	93	6	1
	87/11/2	87	11	2
	80/17/3	80	17	3
	73/23/4	73	23	4
PA410/PA6 unfilled blends	95/5	95	5	0
	85/15	85	15	0
	75/25	75	25	0

**Table 2:** Calorimetric parameters of PA410/PA6 blends.

PA410/PA6 Composition	$T_{m}^{\;1(\text{°}C)}$	$\Delta H_{m}^{\;1(J/g)}$	$T_{c}^{\;2(\text{°}C)}$	$\Delta H_{c}^{\;2(J/g)}$	$T_{m}^{\;3(\text{°}C)}$	$\Delta H_{m}^{\;3(J/g)}$
100/0	257.7	58	226.3	64	255.1	64
95/5	256.7	63	225.3	65	254.4	65
85/15	254.4	57	223.3	62	252.4	63
75/25	253.1	58	221.6	54	250.4	55
0/100	227.4	59	184.6	65	223.1	68

^1^ First heating scan. ^2^ cooling scan from the melt. ^3^ Second heating scan.

**Table 3:** Calorimetric parameters of PA410/CNT NCs.

PA410/CNT Composition	$T_{m}^{\;1(\text{°}C)}$	$\Delta H_{m}^{\;1(J/g)}$	χc (%)	$T_{c}^{\;2(\text{°}C)}$	$\Delta H_{c}^{\;2(J/g)}$
100/0	256.7	59	22	224.3	−39
99/1	257.1	61(62)	23	233.6	−46
98/2	256.7	59(60)	22	234.9	−49
97/3	255.4	59(61)	23	235.9	−51
96/4	257.4	57(59)	22	235.3	−53
95/5	255.7	60(63)	23	235.3	−55
94/6	253.7	55(58)	22	235.6	−55

^1^ First heating scan. ^2^ Cooling from the melt. Values in brackets are normalized with respect to the weight fraction of PA410 in the NCs.

**Table 4:** Calorimetric parameters of PA410/PA6/CNT NCs.

PA410/PA6/CNT Composition	PA6 wt %	$T_{m}^{\;1(\text{°}C)}$	$\Delta H_{m}^{\;1(J/g)}$	$T_{c}^{\;2(\text{°}C)}$	$\Delta H_{c}^{\;2(J/g)}$
100/0/0	0	257.1	58	224.9	−37
96.6/2.9/0.5	2.9	253.7	63(63)	232.9	−46
93/6/1	6	256.1	56(57)	232.9	−51
87/11/2	11	252.1	59(60)	233.6	−50
80/17/3	17	253.7	57(59)	233.3	−51
73/23/4	23	252.1	49(51)	232.9	−49

^1^ First heating scan. ^2^ Cooling from the melt. Values in brackets are normalized to the polymeric phase.

**Table 5:** Percolation thresholds obtained for melt-processed PA-based systems in the present and other works.

Nanocomposite	Processing Method and Conditions	P_c_ (%)	Reference
PA410/CNT	Twin-screw extruder 270 °C, 200 rpm	3.98	Present study
PA410/PA6/CNT (PA6-based masterbatch)	Twin-screw extruder 270 °C, 200 rpm	0.65	Present study
PA6/CNT	Twin-screw mini-extruder 240 °C, 45 rpm	0.4	[33]
PA6/CNT	Twin-screw extruder 260 °C, 150 rpm	2–3	[34]
PA6/CNT	Twin-screw extruder Masterbatch 260 °C, 200 rpm	4–6	[21]
PA12/CNT	Twin-screw microcompounder 260 °C, 250 rpm	1 (low matrix viscosity) 2–2.5 (medium matrix viscosity) 3.5 (high matrix viscosity)	[32]
PA12/CNT	Micro-extruder 190 °C, 180 rpm	1.33	[12]
aPA/CNT	Twin-screw extruder 265, 200 rpm	2.97	[35]

**Table 6:** Young’s modulus, tensile strength and ductility values of PA410/CNT and PA410/PA6/CNT NCs.

	Composition	Young’s Modulus (MPa)	Tensile Strength (MPa)	Strain at Break (%)
PA410/CNT NCs	100/0	2880 ± 30	75.5 ± 0.4	100 ± 30
	99/1	2850 ± 100	74.3 ± 1.2	7 ± 4
	98/2	2860 ± 60	75.6 ± 0.2	7 ± 1
	97/3	2870 ± 40	74.3 ± 1.2	5 ± 1
	96/4	2930 ± 40	75.1 ± 0.7	7 ± 1
	96/5	3010 ± 10	74.5 ± 0.8	5 ± 1
	94/6	3010 ± 50	76.3 ± 0.5	6 ± 0
PA410/PA6/CNT NCs	96.6/2.9/0.5	2930 ± 90	74.3 ± 0.3	8 ± 4
	93/6/1	2850 ± 40	73.5 ± 0.5	5 ± 1
	87/11/2	2930 ± 40	73.8 ± 1.0	6 ± 2
	80/17/3	3040 ± 80	71.8 ± 3.6	4 ± 1
	73/23/4	3180 ± 70	74.1 ± 2.0	3 ± 0

## Supplementary Materials

The following are available online at http://www.mdpi.com/2073-4360/10/9/986/s1, Figure S1: Normalized experimental melting DSC curves of PA6 and PA6/CNT masterbatch, Table S1: Calorimetric parameters of PA6 vs. PA/CNT masterbatch.
