# Supplementary material for: Novel Biobased Polyamide 410/Polyamide 6/CNT Nanocomposites
Source: Polymers (Basel). 2018 Sep 4;10(9):986. doi: 10.3390/polym10090986 (PMC6403815; doi:10.3390/polym10090986)
Supplement: Supplementary file 1 [file polymers-10-00986-s001.zip › polymers-339682-SI.pdf]

Supplementary Material

Novel Biobased Polyamide 410/Polyamide 6/CNT Nanocomposites

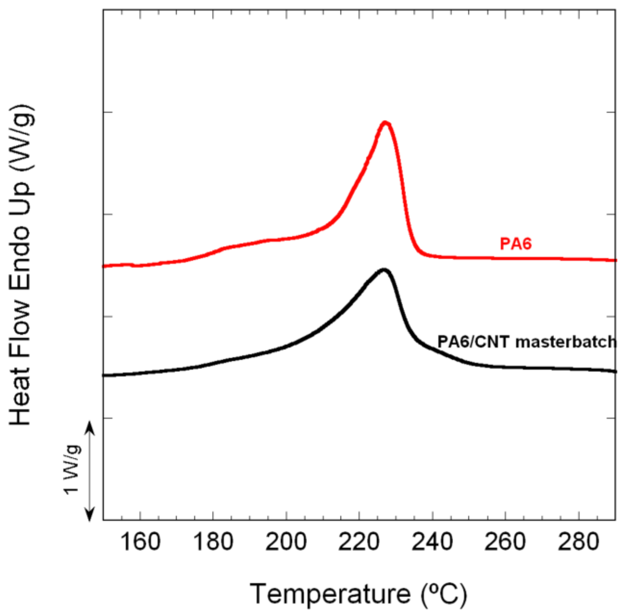

Figure S1. Normalized experimental melting DSC curves of PA6 and PA6/CNT masterbatch.

Table S1. Calorimetric parameters of PA6 vs. PA/CNT masterbatch.

	T_m^3 (ºC)	ΔH_m^3 (J/g)
PA6	227.1	75
PA6/CNT masterbatch	226.7	71(83)

[3]Second heating scan. The value in brackets is normalized to the polymeric phase.
